# Malondialdehyde, an Oxidative Stress Marker in Oral Squamous Cell Carcinoma—A Systematic Review and Meta-Analysis

**DOI:** 10.3390/cimb43020072

**Published:** 2021-08-28

**Authors:** Khadijah Mohideen, Uma Sudhakar, Thayumanavan Balakrishnan, Mazen A. Almasri, Manea Musa Al-Ahmari, Hajar Saeed Al Dira, Malath Suhluli, Alok Dubey, Sheetal Mujoo, Zohaib Khurshid, A. Thirumal Raj, Shankargouda Patil

**Affiliations:** 1 Department of Oral Pathology and Microbiology, Sathyabama Institute of Science and Technology, Sathyabama Dental College and Hospital, Chennai 600119, India; dr.khadijahm@gmail.com (K.M.); thayumds@gmail.com (T.B.); 2 Department of Periodontics and Implantology, Dr. M.G.R. Educational and Research Institute, Thai Moogambigai Dental College and Hospital, Chennai 600095, India; ums_570@yahoo.co.in; 3 Department of Oral Maxillofacial Surgery, King Abdulaziz University, Jeddah 21589, Saudi Arabia; malmasri@kau.edu.sa; 4 Department of Periodontics and Community Dental Sciences, College of Dentistry, King Khalid University, Abha 61421, Saudi Arabia; abudanahmm@gmail.com; 5 College of Dentistry, Jazan University, Jazan 45142, Saudi Arabia; hajar24688@gmail.com; 6 Dental School, Jazan University, Jazan 45142, Saudi Arabia; ml3th2538@gmail.com; 7 Department of Preventive Dental Sciences, College of Dentistry, Jazan University, Jazan 45142, Saudi Arabia; dentaalok@yahoo.com; 8 Division of Oral Medicine & Radiology College of Dentistry, Jazan University, Jazan 45142, Saudi Arabia; sheetalmujoo@yahoo.co.uk; 9 Department of Prosthodontics and Dental Implantology, College of Dentistry, King Faisal University, Al-Ahsa 31982, Saudi Arabia; drzohaibkhurshid@gmail.com; 10 Department of Oral Pathology and Microbiology, Sri Venkateswara Dental College and Hospital, Chennai 600130, India; thirumalraj666@gmail.com; 11 Division of Oral Pathology, Department of Maxillofacial Surgery and Diagnostic Sciences, College of Dentistry, Jazan University, Jazan 45142, Saudi Arabia

**Keywords:** oral squamous cell carcinoma, oral cancer, oxidative stress

## Abstract

Objective: To qualitative and quantitatively review published literature assessing the oxidative stress marker malondialdehyde (MDA) in oral squamous cell carcinoma (OSCC). Methodology: Pubmed (MeSH), Science Direct, Scopus, Web of Science, Willey Online Library, Cochrane, and Cross Reference were searched for studies assessing MDA levels in OSCC samples. Results: From the 1008 articles identified, 849 were excluded based on title and abstract screening due to duplication and irrelevance to the topic of interest. Full-text assessment of the remaining 159 articles led to the inclusion of only 46 articles that satisfied the selection criteria. Of these, only 26 studies had data compatible for quantitative analysis. The MDA levels in OSCC groups are significantly increased (*p* < 0.00001) in plasma, serum, and saliva samples in the majority of the studies evaluated. In contrast, MDA levels in OSCC tissue samples are significantly attenuated (*p* < 0.00001) compared to healthy controls, supported by fewer studies. Conclusions: The augmented MDA levels in plasma, serum, and saliva samples of the OSCC reflect the heightened oxidative stress level accurately. Further studies are required to understand the attenuated MDA levels in the tissue samples of OSCC. Correlation analysis between MDA levels with established clinicopathological prognostic markers could aid in formulating oxidative stress-based prognostication and treatment planning.

## 1. Introduction

Squamous cell carcinoma (SCC) is one of the most common oral malignancies. The incidence of oral cancer varies greatly. The annual worldwide report states the incidence of more than 400,000 new cases of OSCC [1]. Brazil, Central, Eastern Europe, France, and India have the highest reported oral cancer rates worldwide [2].

Various factors are known to play in the etiopathogenesis of oral squamous cell carcinoma. Carcinogenesis may be the interplay of socioeconomic factors and etiological factors such as habitual use of smoking or chewing tobacco, alcohol, oncogenic viral infections, oncogenes, and mutation of tumor suppressor genes. Recent literature showed that young patients who developed oral cancer were non-smokers and not addicted to tobacco/betel nut chewing. An epidemiological study of oral cavity cancers in Iran showed that tongue cancer is the oral cavity’s predominant cancer in non-smokers [3]. Thus, other factors may also be involved in etiopathogenesis. Factors such as phenols, radiation, trauma or sharp teeth, iron deficiency, vitamin A deficiency, syphilis, candidiasis, and a compromised immune status are the suggested other possible causes [4].

The continuous and direct exposure of the oral mucosal cells to the chemical carcinogens of tobacco products such as Polynuclear Aromatic Hydrocarbons (PAH) and nitrosamines tend to induce free radicals/reactive oxygen species (ROS) production [5]. Free radicals are molecules that show an unpaired electron in their external orbit and are therefore highly reactive [6]. Some of the free radicals (ROS) are such as superoxide anion radicals (O^−^_2_), hydroxyl radicals (HO), Hydroperoxyl (HO_2_), peroxyl (ROO.), alkoxyl (RO.), and hydrogen peroxide (H_2_O_2_) [7]. ROS and reactive nitrogen species (RNS) exert beneficial effects on cellular responses and immune function at low or moderate levels. However, at higher levels, ROS produces various pathologies.

Anti-oxidants are cytoprotective chemicals that prevent oxidative damage caused by free radicals [8]. Due to harmful habits, ROS attain higher concentrations which evade or overwhelm the anti-oxidant protective mechanisms provided by anti-oxidants such as superoxide dismutase (SOD), catalase (CAT), glutathione peroxidase (GPx), glutathione reductase (GRx), carotenes, and vitamins of cells and tissues. It results in the depletion of anti-oxidants, which causes the accumulation of ROS and leads to the condition called oxidative stress (OS) [9]. OS induces cell metabolism impairment, including rising intracellular free Ca^2+^ levels and damage of the membrane ion transporters. ROS also facilitates punctual mutations, DNA base oxidations and strand breakage, mutation of tumor suppressor genes, and activation of proto-oncogenes [6,10]. ROS reactions with biological molecules cause damage to lipid bio-membrane, sulfhydryl bonds of proteins and carbohydrates [8]. The bio-membrane lipid peroxidation damage is initiated by abstracting hydrogen from unsaturated fatty acids. The formed free radicals initiate the chain reaction resulting in total degeneration of the cellular membrane, which plays a crucial role in carcinogenesis [10].

Furthermore, the decomposition of these peroxidized lipids are disintegrated quickly and forms reactive carbon compounds, including lipid hydroperoxides (LHP) and malondialdehyde (MDA). These by-products serve as an indicator of lipid peroxidation [11]. These lipid peroxidation products can modulate cell growth and promote tumor progression by activating the signal transduction pathway. In addition, they act as co-carcinogenic agents by expressing their high cytotoxicity [12].

There is a need for quantitation of biomarker expression to assess bio-molecular damage. The measurement of free radicals directly is not reliable due to the concise life of free radicals. Hence, the proposed method of OS evaluation includes the estimation of secondary lipid peroxidation products, such as MDA. Hence, MDA assessment expresses the extent of lipid peroxidation and free radical-mediated oxidative damage. MDA is a three-carbon dialdehyde compound that appears in blood, saliva, serum, tissue, and urine during lipid peroxidation [13]. Hence, the present review aimed to analyze oxidative stress using MDA as a biomarker of lipid peroxidation (LPx) in OSCC patients and compare them with the healthy control group with the help of the available literature.

## 2. Materials and Methods

### 2.1. Protocol and Registration

PRISMA guidelines had been strictly adhered to study selection. The review protocol was registered in the PROSPERO database (CRD42021249182).

### 2.2. Focused Question

Is there any significant difference in the MDA level of biological samples between oral squamous cell carcinoma patients and the control group?

Based on the objective of the present meta-analysis and the research question, the following components were focused:

(i) Population: patients with OSCC
(ii) Exposure or Diagnostic marker: mean and standard deviation value of MDA
(iii) Comparison: between patients with oral squamous cell carcinoma and healthy subjects
(iv) Outcome: assessment of MDA in various biological samples of patients with OSCC
(v) Study: identify related cross-sectional and case-controlled studies investigating the status of MDA in OSCC and control from 1999 to 2020.

### 2.3. Electronic Search Identification

Electronic databases, including PubMed (MeSH), Science Direct, Scopus, Web of Science, Willey Online Library, Cochrane, and Cross Reference, were searched for published articles addressing oxidative stress in oral squamous cell carcinoma using MDA assay between the years 1999–2020. The following keywords, ‘oral squamous cell carcinoma,’ ‘oxidative stress,’ and ‘Malondialdehyde was employed.’

### 2.4. Screening for Relevance

Articles discussing oxidative stress in OSCCwere identified and shortlisted based on the titles and abstracts screening for relevance and duplication.

### 2.5. Inclusion Criteria

(a) Studies discussed the oxidative status of OSCC using lipid peroxidation marker-Malondialdehyde (MDA);
(b) Studies involving various biological samples and expressed the MDA data in mean, standard deviation along with *p*-value;
(c) Papers provided sufficient data to allow comparison of OSCC and control groups.

### 2.6. Exclusion Criteria

1. Articles with the unmatched objective and abstract;
2. Being literature reviews and systematic reviews;
3. Studies used other oxidative stress markers as a marker of evaluation;
4. The works provided inadequate data for the comparison between control and OSCC groups;
5. Studies related to head and neck squamous cell carcinoma

### 2.7. Retrieval of Full-Text Articles and Evaluation

K.M., U.S., and T.B. screened the titles/abstracts of all the studies and excluded studies at high risk of bias from the evidence synthesis based on pre-specified criteria. K.M., S.P., and A.T.R., have independently screened each included study’s full texts. K.M., M.M.A.A, M.A.A, H.S.A.D, Z.K., and A.T.R., have checked and discussed the relevant factors considered in each included study. After assessing all the particulars, the authors have considered the articles for eligibility criteria. The authors resolved disagreements by consensus. Finally, K.M., U.S., and S.P., have performed the data collection procedure.

### 2.8. Data Extraction

The extracted data from full-text articles were author, publication year, age groups, sample size, MDA measurements in OSCC, and control group expressed as the mean and standard deviation along with specific units. Collected data were tabulated separately in a specified format.

### 2.9. Statistical Analysis

The Forest plot was derived using the mean difference, and standard mean difference method to carry out a meta-analysis using comprehensive meta-analysis software version 3 (Biostat Inc. Englewood, NJ, USA). The overall mean difference or standardized mean difference value of MDA in OSCC was analyzed at a 95% confidence interval (CI). A random-effects model was used in the analysis due to the presence of significant heterogeneity. The articles, which expressed the MDA levels in similar units in each sample, only were included in the meta-analysis.

## 3. Results

**Figure 1:**
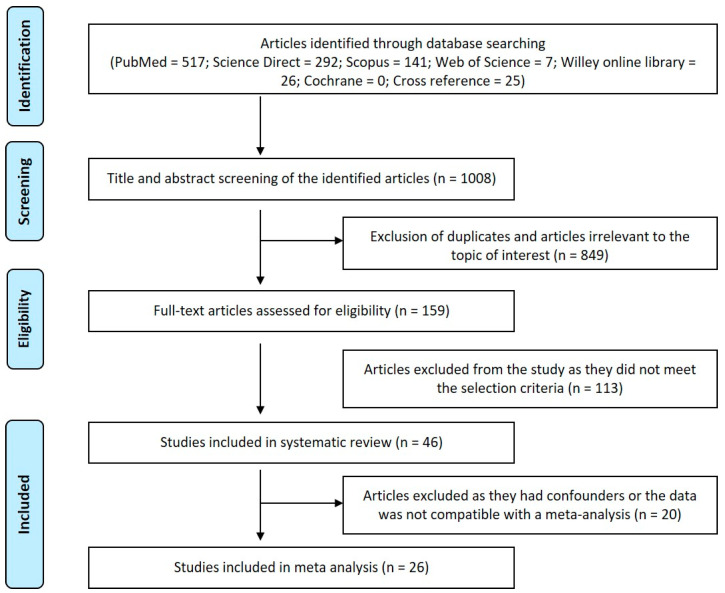
Prisma Flow Chart—Study Selection.

Pubmed search yielded 517 papers; Science direct search yielded 292 papers; Scopus search yielded 141 papers; Web of Science yielded seven papers; Willey online library yielded 26 papers, and Cross-reference search yielded 25 papers. After search refinement, 849 articles were excluded due to unmatched titles and abstracts, including four duplicated data reports and one animal study. After extraction of these articles, 159 articles had their titles relevant to the present work. Full-text was retrieved for the screened articles. Articles with un-matched objectives (*n* = 84), systematic reviews (*n* = 1), critical reviews (*n* = 2), reviews (*n* = 25) and letter to the editor (*n* = 1) were excluded. Forty-six articles with matched objectives were included in the systematic review. Only 26 articles had data compatible for a meta-analysis (Figure 1).

Newcastle-Ottawa quality assessment scale was employed to grade the quality of included studies in the systematic review (Table 1). Collected MDA assessment data along with other findings of included articles in various biological samples were tabulated (Table 2). Few studies compared the MDA level concerning clinical stages of OSCC in various samples (Table 3) and changes in varying histopathological grades (Table 4). The analysis of MDA levels according to different clinical stages and histopathological grades could not be performed due to the scarcity of the reported studies.

**Figure 2:**
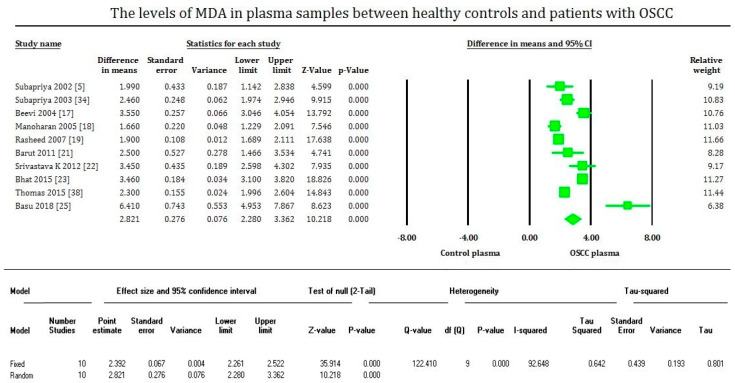
Forest plot shows mean difference estimates with 95% confidence intervals representing differences in plasma levels of MDA between the oral squamous cell carcinoma group and healthy controls.

**Figure 3:**
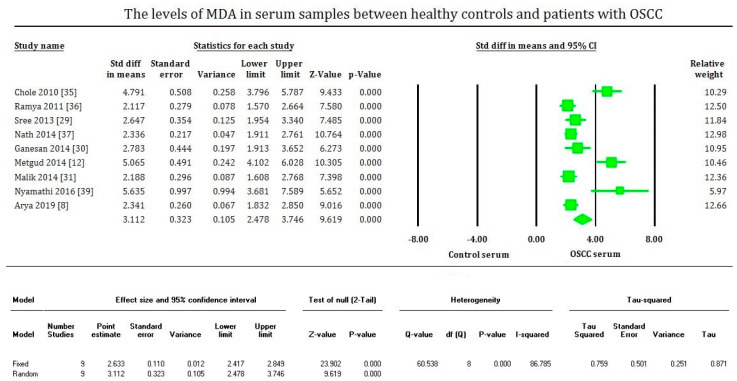
Forest plot shows mean difference estimates with 95% confidence intervals representing differences in serum levels of MDA between oral squamous cell carcinoma group and healthy controls.

**Figure 4:**
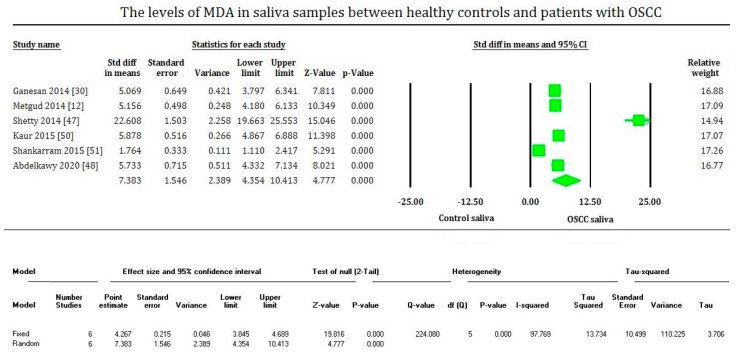
Forest plot shows mean difference estimates with 95% confidence intervals representing differences in salivary levels of MDA between oral squamous cell carcinoma group and healthy controls.

**Figure 5:**
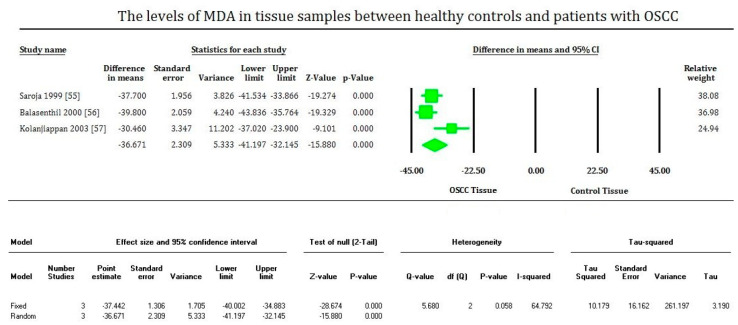
Forest plot shows mean difference estimates with 95% confidence intervals representing differences in tissue levels of MDA between oral squamous cell carcinoma group and healthy controls.

MDA levels are significantly increased (*p* < 0.00001) in OSCC in the plasma, serum, and saliva samples of most of the studies evaluated. On the contrary, MDA levels of tissue samples are significantly decreased (*p* < 0.00001) in OSCC compared to healthy tissues, supported only by fewer studies. The plasma samples showed an overall mean difference of 2.81 with 95% CI (2.280–3.362) [Figure 2]. The serum samples showed an overall standard mean difference of 3.112 with 95% CI (2.478–3.746) [Figure 3]. The saliva samples showed an overall standard mean difference of 7.383 with 95% CI (4.354–10.413) [Figure 4]. The tissue samples showed an overall mean difference of −36.671 with 95% CI (−41.197 to −32.145) [Figure 5].

The meta-analysis presented high heterogeneity, reflected by the I^2^ values 92.648, 86.785, 97.769, and 64.792 of Figure 2, Figure 3 and Figure 4, respectively. The different methodologies utilized to measure MDA levels could be the reason for the high heterogeneity.

## 4. Discussion

Lipid peroxidation is a sequential reaction providing a constant supply of free radicals that initiate further peroxidation and free radicals accumulation, resulting in OS [77]. The endogenous formation of MDA during lipid peroxidation serves as a suitable biomarker of endogenous DNA damage [12]. MDA interacts with cellular DNA and forms MDA deoxyguanosine (M1-dG), a DNA-MDA covalently bonded adduct, resulting in DNA damage that causes interference in repair [78]. This mutagenic transformation within the DNA alters their chemical behavior and possibly contributing to carcinogenesis. These reactive aldehydes (MDA) also bind to membrane proteins. They cause profound changes in their function, tonicity, permeability, rigidity, structural integrity, and enhancing neoplastic transformation of the affected tissues. Thus, the developed OS affects the cell membrane’s essential constituents, which ultimately increases cell proliferation and actively influences cancer initiation, promotion, and progression [79].

The present systematic review included the research articles that involve 1307 patients diagnosed with OSCC and 1217 healthy volunteers for MDA analysis in various biological samples.

Previous studies demonstrated enhanced lipid peroxidation and malondialdehyde in patients with OSCC. The included studies had found a statistically significant increase in plasma or serum MDA levels in OSCC patients compared with controls (*p* < 0.001) [8,12,19,20,21,22,24,30,32,35,36,37,39,40,43,46,47,49,65]. Similarly, other studies also observed a significant rise compared with the control group (*p* < 0.05) [8,17,25,31,34,44,52,53,67]. Other studies also reported MDA rise in erythrocytes with statistical significance (*p* < 0.001) [20,26], (*p* < 0.01) [38] and (*p* < 0.05) [5,17,27]. On the contrary, one report did not show any change in blood MDA level in OSCC patients than in control [28]. In the present meta-analysis, the plasma samples showed an overall mean difference of 2.79 with a 95% CI (2.26–3.32). The serum samples showed an overall mean difference of 7.43 with 95% CI (5.99–8.87). The serological changes are consistent even though they are secondary to the tissue changes taking place anywhere in the body. A few studies had also reported higher salivary MDA levels in OSCC compared with healthy subjects with statistical significance (*p* < 0.001) [12,29,33,35,36,54] and (*p* < 0.05) [23,41,42,50]. However, three included studies expressed that the increase in the MDA level in saliva and mitochondria was insignificant (*p* > 0.05) [48,51]. In the present work, the saliva samples showed an overall mean difference of 0.91 with a 95% CI (0.63–1.18). The increased levels could be due to the disintegration of polyunsaturated fatty acids of bio-membranes due to oxidative lipid damage [19]. The evaluation of tissue MDA level also showed a rise in OSCC patients than the control group with statistical significance (*p* < 0.001) [36], (*p* < 0.01) [38], and (*p* < 0.05) [27]. On the contrary, few authors differently reported the tissue MDA levels of the OSCC group [5,14,16,18,45]. Their studies in tissue displayed a decrease in mean MDA level in OSCC patients compared to the control group with statistical significance. (*p* < 0.001) [55,56,57,58] and (*p* < 0.05) [5]. In the present analysis, the tissue samples showed an overall mean difference of −37.08 with 95% CI (−41.25 to −32.92). The decrease in MDA levels observed in the tumor tissues of oral cancer patients reflects a decreased susceptibility of oral tumor tissue to lipid peroxidation. Srivastava 2016 et al. hypothesized that serum biology compared to tissue poses a considerable threat and produces free radicals in excess amounts [45]. They are readily diffused inside the cell to cause various mutations, favoring carcinogenesis. On the other hand, the tissue produces a relatively lesser amount of free radicals and, at the same time, is capable of counteracting them with the available enzymes. Therefore, Srivastava et al. stated that the external environment and the internal factors influence the selective growth of the tumor cells [45].

There is a gradual increase in the MDA level in plasma and erythrocyte when the clinical stage of OSCC advances on further analysis. According to severity, the difference in the rise of plasma MDA levels between the advancing stages was statistically significant within all the clinical grades (*p* < 0.01) [20] and (*p* < 0.001) [32]. Arya et al. observed a significant increase in serum MDA value from T1 to T3 group, and the *p*-value was <0.05 [8]. Therefore, a positive relationship between serum MDA level and tumor size was found. The authors stated that lipid peroxidation increases with the disease severity. Therefore, serological levels are reflecting the extent of tissue injury [24].

In contrast, Babiuch et al. observed decreasing salivary MDA value when the tumor progresses from T1 to T4 in size, statistically insignificant [51]. Two reported studies in tissue displayed a decreasing mean MDA level when the clinical stage of OSCC advances, which is statistically significant in one study (*p* < 0.01) [18] and insignificant in another report (*p* > 0.05) [45].

Few studies reported an increase in plasma and serum MDA level when histological grades of the disease advance with statistical significance (*p* < 0.001) [40] and (*p* < 0.01) [34]. On the contrary, three studies stated that lipid peroxidation level was inversely proportional to the degree of differentiation of OSCC as the grade advances. However, the change was statistically non-significant (*p* > 0.05) [8,12,25]. These results correlated with Salzman et al. 2009, who showed a negative correlation of MDA and tumor grade [80]. Thus, there was no definitive correlation pattern in lipid peroxidation between degrees of differentiation of malignant oral lesions. The expression of serum MDA levels in different histopathological grades exhibits a complex relationship. The present meta-analysis showed the MDA levels are significantly increased (*p* < 0.00001) in OSCC in all the samples of plasma, serum, and saliva except the tissue samples where MDA levels are significantly decreased (*p* < 0.00001) in OSCC compared to healthy tissues. The tissue-level changes with advancing clinical stages of the tumors were also very poorly explored. The authors used different methodologies to assess MDA levels in various biological samples [55,57,58,59,60,61,62,63,64,66,68,69,70,71,72]. The reported studies utilized different clinical staging systems [73,81] and histopathological grading systems [74,75,76] to categorize the OSCC group patients. It will be worthwhile if future studies consider these facts in the MDA assessment of the OSCC group to evaluate the effect of oxidative stress on tumors. Although various treatments have been proposed to manage this type of cancer, its aggressiveness and ability to metastasize make this cancer one of the most difficult to treat, so early diagnosis is crucial when facing this condition [82,83]. Therefore, the studies evaluating the OS will improve the understanding of the anti-oxidant enzyme activity in the early diagnosis and treatment of oral cancer [15].

## 5. Conclusions

The oxidant/anti-oxidant equilibrium is a critical step toward developing more effective strategies for prevention, early detection, and treatment of oral cancer. Estimating lipid peroxidation by-products in the OSCC group could assess the degree of oxidative stress-related tissue injury. Therefore, the assay of malondialdehyde level in oral cancer may be helpful to evaluate the disease severity for both preventive and clinical intervention. Most studies revealed the significant elevation of malondialdehyde levels in oral squamous cell carcinoma patients than healthy controls. Therefore, there is a requirement of large-scale studies with better-matched controls and equal distribution of samples among different clinical stages and histological grades of OSCC to conclude MDA as a potential biomarker for oxidative stress and valid prognostic marker of OSCC.

## Figures and Tables

**Table 1:** New Castle Ottawa Scale for studies included in the Systematic Review.

	Selection				Comparability		Exposure				
Study (Reference Number)	Case Definition	Case Representativeness	Control Selection	Control Definition	Matching Known Confounding Factor	Matching Potential Confounding Factor	Secure Patient Records	Interviewer Blinded to Cases and Control	Similarityin Case and Control Ascertainment	Non-Response Rate	Total Stars
Saroja et al. 1999 [14]	*	*	*	*	*	-	*	-	*	*	8
Sabitha et al. 1999 [15]	*	*	*	*	*	-	-	-	*	*	7
Balasenthil et al. 2000 [16]	*	*	*	*	*	-	*	-	*	-	7
Subapriya et al. 2002 [5]	*	*	*	*	*	*	*	-	*	-	8
Subapriya et al. 2003 [17]	*	*	*	*	*	-	*	-	-	*	7
Kolanjiappan et al. 2003 [18]	*	*	*	*	*	-	*	-	-	*	7
Beevi et al. 2004 [19]	*	*	*	*	*	*	*	-	*	-	8
Manoharan et al. 2005 [20]	*	*	*	*	*	*	*	-	*	*	9
Khanna et al. 2005 [21]	*	*	*	*	*	*	*	-	*	*	9
Rasheed et al. 2007 [22]	*	*	*	*	*	-	*	-	*	*	8
Rai B et al. 2008 [23]	*	*	*	*	*	-	*	-	*	-	7
Bathi et al. 2009 [24]	*	*	*	*	*	*	*	-	*	*	9
Chole et al. 2010 [25]	*	*	*	*	*	*	-	-	*	*	8
Raghavendra et al. 2010 [26]	*	*	*	*	*	-	*	-	*	*	8
Gokul et al. 2010 [27]	*	*	*	*	*	*	*	-	*	-	8
Burlakova et al. 2010 [28]	*	*	*	*	*	-	*	-	*	*	8
Arathi et al. 2010 [29]	*	*	*	*	*	-	*	-	*	*	8
Barut et al. 2011 [30]	*	*	*	*	*	*	*	-	*	*	9
Ramya et al. 2011 [31]	*	*	*	*	*	*	-	-	*	*	8
Srivastava K et al. 2012 [32]	*	*	*	*	*	-	-	-	*	*	7
Sree et al. 2013 [33]	*	*	*	*	*	-	*	-	-	*	7
Nath et al. 2014 [34]	*	*	*	*	-	-	*	-	-	*	6
Metgud et al. 2014 [12]	*	*	*	*	*	-	*	-	*	*	8
Rasool et al. 2014 [35]	*	*	*	*	*	-	*	-	-	*	7
Ganesan et al. 2014 [36]	*	*	*	*	*	-	*	-	*	*	8
Malik et al. 2014 [37]	*	*	*	*	*	-	*	-	*	*	8
Huo et al. 2014 [38]	*	*	*	*	*	-	-	-	*	*	7
Shetty et al. 2014 [33]	*	*	*	*	*	-	*	-	*	*	8
Bhat et al. 2015 [39]	*	*	*	*	*	-	*	-	-	*	7
Rai S et al. 2015 [40]	*	*	*	*	-	*	*	-	-	*	7
Thomas et al. 2015 [38]	*	*	*	*	*	-	*	-	-	*	7
Kaur et al. 2015 [41]	*	*	*	*	*	-	*	-	*	*	8
Shankarram et al. 2015 [42]	*	*	*	*	-	-	*	-	-	*	6
Mishra et al. 2016 [43]	*	*	*	*	-	*	*	-	*	-	7
Nyamathi et al. 2016 [44]	*	*	*	*	*	-	*	-	*	-	7
Srivastava K et al. 2016 [45]	*	*	*	*	*	*	*	-	*	*	9
Verma et al. 2017 [46]	*	*	*	*	*	-	*	-	*	*	8
Madhulatha et al. 2017 [47]	*	*	*	*	-	*	*	-	-	*	7
Banerjee et al. 2017 [48]	*	*	*	*	*	*	*	-	*	*	9
Basu et al. 2018 [49]	*	*	*	*	*	-	-	-	-	*	6
Arya et al. 2019 [8]	*	*	*	*	*	-	*	-	*	*	8
Sabarathnam et al. 2019 [50]	*	*	*	*	-	-	-	-	*	*	6
Babiuch et al. 2019 [51]	*	*	*	*	*	*	*	-	*	*	9
Shahi et al. 2020 [52]	*	*	*	*	*	*	*	-	*	*	9
Oswal et al. 2020 [53]	*	*	*	*	*	-	*	-	-	*	7
Abdelkawy et al. 2020 [54]	*	*	*	*	*	-	*	-	*	*	8

**Table 2:** The levels of MDA in various biological samples between healthy controls and patients with OSCC of studies included in the qualitative synthesis.

Author			OSCC			Control			Method
	Sample	Unit	Mean	Std. Dev	Sample Size	Mean	Std. Dev	Sample Size	
Saroja 1999 [14] *	Ti	nmol/100 mg protein	86.56	8.03	33	124.3	7.86	33	Ohkawa et al. [55]
Sabitha 1999 [15]	Se	ηmol/mL	0.598	0.169	12			12	Suematsu et al. [56]
Balasenthil 2000 [16] *	Ti	nmol/100 mg protein	85.5	4.4	10	125.3	4.8	10	Ohkawa et al. [55]
Subapriya 2002 [5]	Ti	nmol/100 mg protein	97.84	9.32	24			24	Ohkawa et al. [55]
Subapriya 2002 [5] *	Pl	nmol/mL	6.37	1.12	24	4.38	1.8	24	Yagi et al. [57]
Subapriya 2002 [5]	Er	pm/mg Hg	1.98	0.21	24	1.11	0.13	24	Donnan et al. [58]
Subapriya 2003 [17] *	Pl	nmol/mL	6.27	0.72	6	3.81	0.35	12	Yagi et al. [57]
Subapriya 2003 [17]	Er	mg/dL	39.44	3.6	6	34.61	3.3	12	Buege et al. [59]
Kolanjiappan 2003 [18] *	Ti	nmol/100 mg protein	93.4	10.5	48	123.9	14.5	16	Ohkawa et al. [55]
Beevi 2004 [19] *	Pl	nmol/mL	5.57	0.97	15	2.02	0.23	15	Draper et al. [60]
Manoharan 2005 [20] *	Pl	nmol/mL	3.75	0.87	48	2.09	0.17	16	Yagi et al. [57]
Manoharan 2005 [20]	Er	pm/mg Hb	3.35	0.43	48	2.43	0.17	16	Donnan et al. [58]
Manoharan 2005 [20]	Er memb	nmol/mg protein	0.62	0.2	48	0.34	0.06	16	Donnan et al. [58]
Khanna 2005 [21]	Se	nmol/L	0.67	0.57	20	0.321	0.06	20	Bergmeyer et al. [61]
Rasheed 2007 [22] *	Pl	nmol/mL	4.16	0.47	24	2.26	0.24	24	Draper et al. [60]
Rai B 2008 [23]	Sa	ng/mL	5.23	0.41	12	3.415	0.44	30	Buege et al. [59]
Bathi 2009 [24]	Pl		3.543		30	2.517		30	Jain et al. [62]
Chole 2010 [25] *	Se	ηmol/mL	14.34	1.43	30	5.107	2.32	30	Ohkawa et al. [55]
Raghavendra 2010 [26]	Er	nmol/mL	7.22	1.52	25	4.379	0.97	25	Stocks et al. [63]
Gokul 2010 [27]	Er	nmol/g Hg	159.8	36.4	18	139.4	22.3	25	Ohkawa et al. [55]
Gokul 2010 [27]	Ti	nmol/mg protein	1.12	0.76	18	0.68	0.33	18	Ohkawa et al. [55]
Burlakova 2010 [28]	Er	µmol/10^6^ Er	3.5	0.52	50	3.92	1.06	54	Valenzuela et al. [64]
Arathi 2010 [29]	Sa	nmol/L	0.017	0.01	25	0.002	0	25	Stocks et al. [63]
Barut 2011 [30] *	Pl	nmol/mL	7.4	2.55	29	4.9	1.25	29	Buege et al. [59]
Ramya 2011 [31] *	Se	nmol/mL	1.79	0.29	40	1.16	0.31	40	Ohkawa et al. [55]
Srivastava K 2012 [32] *	Pl	nmol/mL	5.5	1.7	20	2.05	0.94	20	Yagi et al. [57]
Sree 2013 [65] *	Se	nmol/mL	5.32	1.12	30	3.18	0.23	30	Ohkawa et al. [55]
Nath 2014 [34] *	Se	nmol/mL	55.04	13.7	120	27.43	2.62	45	Ohkhawa et al. [55]
Metgud 2014 [12] *	Se	nmol/mL	6.02	0.43	40	2.93	0.79	30	Okhawa et al. [55]
Metgud 2014 [12] *	Sa	nmol/mL	0.32	0.03	40	0.2	0.01	30	Ohkawa et al. [55]
Rasool 2014 [35]	Pl	µmol/mL	4.55	1.48	30	3.15	0.58	10	Spectrophotometry
Rasool 2014 [35]	Sa	µmol/mL	0.54	0.25	30	0.19	0.02	10	Spectrophotometry
Ganesan 2014 [36] *	Se	nmol/mL	1.824	0.55	20	0.712	0.13	20	Okhawa et al. [55]
Ganesan 2014 [36] *	Sa	nmol/mL	1.007	0.16	20	0.349	0.09	20	Okhawa et al. [55]
Ganesan 2014 [36]	Ti	nmol/mL	1.115	0.12	20	0.59	0.13	20	Ohkawa et al. [55]
Malik 2014 [37] *	Se	nmol/mL	18.72	5.56	45	8.5	2.83	30	Ohkawa et al. [55]
Huo 2014 [38]	Er	nmol/g Hg	164		25	144		25	Ohkawa et al. [55]
Huo 2014 [38]	Ti	nmol/mg protein	3		15	0.8		15	Ohkawa et al. [55]
Shetty 2014 [33] *	Sa	nmol/mL	0.931	0.03	50	0.181	0.03	65	TBA-TCA
Bhat 2015 [39] *	Pl	nmol/mL	5.58	0.98	30	2.12	0.23	30	Draper et al. [60]
Rai S 2015 [40]	Pl		13.16	0.55	20	2.92	0.36	20	Satoh et al. [66]
Thomas 2015 [67] *	Pl	nmol/mL	5.2	0.49	20	2.9	0.49	20	Mahfouz et al. [68]
Kaur 2015 [41] *	Sa	nmol/mL	1	0.21	40	0.08	0.07	40	Buege et al. [59]
Shankaram 2015 [42] *	Sa	nmol/mL	5.94	0.9	25	4.43	0.81	25	NWLSS NWK
Mishra 2016 [43]	Se		14.15	0.47	20	2.92	0.36	20	Satoh et al. [66]
Nyamathi 2016 [44] *	Se	nmol/mL	13.22	2.4	10	3.4	0.56	10	Satoh et al. [66]
Srivastava K 2016 [45]	Ti	nmol/mL	87.53	2.65	20	127.9	2.97	20	Ohkawa et al. [55]
Verma 2017 [46]	Pl	µmol/mL	3.38	0.14	20	2.45	0.13	20	Sinnhuber et al. [69]
Madhulatha 2017 [47]	Se		4.34	1.69	25	2.97	1.09	25	Gavino et al. [70]
Bannerjee 2017 [48]	Mi	nmol/mg protein	6.093	0.76	60	1.49	0.19	20	Ogura et al. [71]
Basu 2018 [49] *	Pl	nmol/mL	20.35	4.15	30	13.94	2.51	50	Yagi et al. [57]
Arya 2019 [8] *	Se	nmol/mL	57	26.8	50	10.5	8.43	50	Oxitek Assay kit
Sabarathinam 2019 [50]	Sa	µg/mg	2.7	0.15	10	0.9	0.05	15	Spectrophotometry
Babiuch 2019 [51]	Sa	nmol/L	8.58	6.23	20	2.32	5.36	20	Kit-My BioSource (USA)
Shahi 2020 [52]	Pl	µmol/mL	0.82	0.7	25	0.39	0.2	45	Nair et al. [72]
Oswal 2020 [53]	Se		13.4		25	2.91		30	
Abdelkawy 2020 [54] *	Sa	nmol/mL	3.62	0.61	20	1.03	0.19	20	ELISA kit Sun Long Biotech

Abbreviations: Ti—Tissue, Se—Serum, Pl—Plasma, Er—Erythrocyte, Er memb—Erythrocyte Membrane, Mi—Mitochondria, Sa—Saliva, Std. Dev—Standard Deviation *—Studies used for Meta-analysis.

**Table 3:** The levels of MDA in various samples of patients with different clinical stages of OSCC.

Author				OSCC Stage II		OSCC Stage III		OSCC Stage IV			
	Sample	Sample Size	Unit	Mean	Std Dev	Mean	Std Dev	Mean	Std Dev	Stat Sig	Clinical Stage Criteria
Manoharan 2005 [20]	Pl	48	nmol/mL	2.88	0.24	3.54	0.88	4.83	1.51	<0.01	Sobin et al. (UICC) [73]
Srivastava K 2012 [32]	Pl	20	nmol/mL	3.2	1.09	5.42	0.53	7.12	0.35	<0.001	TNM
Manoharan 2005 [20]	Er	48	pm/mg Hb	2.67	0.21	3.35	0.91	4.02	0.16	<0.01	Sobin et al. (UICC) [73]
Manoharan 2005 [20]	Er memb	48	nmol/mg protein	0.41	0.08	0.6	0.24	0.87	0.28	<0.01	Sobin et al. (UICC) [73]
Kolanjiappan 2003 [18]	Ti	48	nmol/100 mg protein	105.4	11.1	94.3	10.4	80.51	9.96	<0.01	AJCC 1992 [74]
Srivastava K 2016 [32]	Ti	20	nmol/mL	89.64	0.66	88.1	1.78	85.72	2.97	> 0.05	TNM
Banerjee 2017 [48]	Mi	60	nmol/mg protein	8.25	0.841	3.3	0.743	5.33	0.659	0.986	TNM
			T1		T2		T3		T4		
Babiuch 2019 [51]	Sa	20	10.5	8.22	8.7	5.85	8.59	7.57	4.16	0.73	T Stage

Abbreviations: Ti—Tissue, Pl—Plasma, Er—Erythrocyte, Er memb—Erythrocyte Membrane, Mi—Mitochondria, Sa—Saliva, Std. Dev—Standard Deviation, Stat Sig—Statistical Significance.

**Table 4:** The levels of MDA in various samples of patients with different histopathological grades of OSCC.

Author				OSCC (WD)		OSCC (MD)		OSCC (PD)			
	Sample	Sample SIZE	Unit	Mean	Std Dev	Mean	Std Dev	Mean	Std Dev	Stat Sig	Histological Grade Criteria
Rai S 2015 [40]	Pl	20		12.98	0.67	13.34	0.42	-	-	<0.001	Akhter et al. [75].
Chole 2010 [25]	Se	30	ηmol/mL	14.81	1.54	14.68	1.8	13.2	0.54	>0.05	
Nath 2014 [34]	Se	120	nmol/mL	39.11	9.031	49.6	6.53	76.4	25.68	<0.01	Anneroth et al. [76]
Metgud 2014 [12]	Se	40	nmol/mL	6.12	0.36	5.92	0.49	-	-	> 0.05	
Arya 2019 [8]	Se	50	nmol/mL	59.81	26.9	53.55	28.13	33.79	1.7	>0.05	Bryne et al. [74]
Metgud 2014 [12]	Sa	40	nmol/mL	0.33	0.035	0.325	0.024	-	-	>0.05	

Abbreviations: Se—Serum, Pl—Plasma, Sa—Saliva, WD—Well Differentiation, MD—Moderate Differentiation, PD—Poor Differentiation, Std. Dev—Standard Deviation, Stat Sig—Statistical Significance.

## References

[1] Zanaruddin S.N.S., Yee P.S., Hor S.Y., Kong Y.H., Ghani W.M.N.W.A., Mustafa W.M.W., Zain R.B., Prime S.S., Rahman Z.A.A., Cheong S.-C. (2013). Common Oncogenic Mutations Are Infrequent in Oral Squamous Cell Carcinoma of Asian Origin. PLoS ONE.

[2] Chaturvedi A.K., Anderson W.F., Lortet-Tieulent J., Curado M.P., Ferlay J., Franceschi S., Rosenberg P.S., Bray F., Gillison M.L. (2013). Worldwide Trends in Incidence Rates for Oral Cavity and Oropharyngeal Cancers. J. Clin. Oncol..

[3] Saedi B., Razmpa E., Ghalandarabadi M., Ghadimi H., Saghafi F., Naseri M. (2012). Epidemiology of oral cavity cancers in a country located in the esophageal cancer belt: A case control study. Iran. J. Otorhinolaryngol..

[4] Glick M., Feagans W. (2015). Burket’s Oral Medicine and Diagnosis.

[5] Subapriya R., Kumaraguruparan R., Ramachandran C.R., Nagini S. (2002). Oxidant-antioxidant status in patients with oral squamous cell carcinomas at different intraoral sites. Clin. Biochem..

[6] Flint P.W., Haughey B.H., Robbins K.T., Thomas J.R., Niparko J.K., Lund V.J., Lesperance M.M. (2010). Cummings Otolaryngology—Head and Neck Surgery E-book.

[7] Lobo V., Patil A., Phatak A., Chandra N. (2010). Free radicals, antioxidants and functional foods: Impact on human health. Pharmacogn. Rev..

[8] Arya H., Ganvir S.M., Begde D.N., Passi A.D. (2019). Comparative Evaluation of Serum Malondialdehyde (MDA) Level in Oral Submucous Fibrosis and Oral Squamous Cell Carcinoma. J. Clin. Diagnostic Res..

[9] Katakwar P., Metgud R., Naik S., Mittal R. (2016). Oxidative stress marker in oral cancer: A review. J. Cancer Res. Ther..

[10] Gurudath S., Ganapathy K.S., Pai A., Ballal S., Asha M.L. (2012). Estimation of superoxide dismutase and glutathione peroxidase in oral submucous fibrosis, oral leukoplakia and oral cancer—A comparative study. Asian Pac. J. Cancer Prev..

[11] D’souza D., Subhas B.G., Shetty S.R., Balan P. (2012). Estimation of serum malondialdehyde in potentially malignant disorders and post-antioxidant treated patients: A biochemical study. Contemp. Clin. Dent..

[12] Metgud R., Bajaj S. (2014). Evaluation of salivary and serum lipid peroxidation, and glutathione in oral leukoplakia and oral squamous cell carcinoma. J. Oral Sci..

[13] Lieberman M.A., Marks A.D. (2013). Oxygen toxicity and free radical injury. Marks’ Basic Medical Biochemistry: A Clinical Approach.

[14] Saroja M., Balasenthil S., Nagini S. (1999). Tissue lipid peroxidation and glutathione-dependent enzyme status in patients with oral squamous cell carcinoma. Cell Biochem. Funct..

[15] Sabitha K.E., Shyamaladevi C.S. (1999). Oxidant and antioxidant activity changes in patients with oral cancer and treated with radiotherapy. Oral Oncol..

[16] Balasenthil S., Saroja M., Ramachandran C.R., Nagini S. (2000). Of humans and hamsters: Comparative analysis of lipid peroxidation, glutathione, and glutathione-dependent enzymes during oral carcinogenesis. Br. J. Oral Maxillofac. Surg..

[17] Subapriya R., Kumaraguruparan R., Nagini S., Thangavelu A. (2003). Oxidant-antioxidant status in oral precancer and oral cancer patients. Toxicol. Mech. Methods.

[18] Kolanjiappan K., Ramachandran C., Manoharan S. (2003). Biochemical changes in tumor tissues of oral cancer patients. Clin. Biochem..

[19] Beevi S.S.S., Rasheed A.M.H., Geetha A. (2004). Evaluation of oxidative stress and nitric oxide levels in patients with oral cavity cancer. Jpn. J. Clin. Oncol..

[20] Manoharan S., Kolanjiappan K., Suresh K., Panjamurthy K. (2005). Lipid peroxidation & antioxidants status in patients with oral squamous cell carcinoma. Indian J. Med. Res..

[21] Khanna R., Thapa P.B., Khanna H.D., Khanna S., Khanna A.K., Shukla H.S. (2005). Lipid peroxidation and antioxidant enzyme status in oral carcinoma patients. Kathmandu Univ. Med. J..

[22] Rasheed M.H., Beevi S.S., Geetha A. (2007). Enhanced lipid peroxidation and nitric oxide products with deranged antioxidant status in patients with head and neck squamous cell carcinoma. Oral Oncol..

[23] Rai B., Kharb S., Jain R., Anand S.C. (2008). Salivary lipid peroxidation product malonaldehyde in pre-cancer and cancer. Adv. Med. Dent. Sci..

[24] Bathi R.J., Rao R., Mutalik S. (2009). GST null genotype and antioxidants: Risk indicators for oral pre-cancer and cancer. Indian J. Dent. Res..

[25] Chole R.H., Patil R.N., Basak A., Palandurkar K., Bhowate R. (2010). Estimation of serum malondialdehyde in oral cancer and precancer and its association with healthy individuals, gender, alcohol, and tobacco abuse. J. Cancer Res. Ther..

[26] Raghavendra U., D’Souza V., D’Souza B. (2010). Erythrocyte malondialdeyde and antioxidant status in oral squamous cell carcinoma patients and tobacco chewers/smokers. Biomed. Res..

[27] Gokul S., Patil V.S., Jailkhani R., Hallikeri K., Kattappagari K.K. (2010). Oxidant-antioxidant status in blood and tumor tissue of oral squamous cell carcinoma patients. Oral Dis..

[28] Burlakova E.B., Zhizhina G.P., Gurevich S.M., Fatkullina L.D., Kozachenko A.I., Nagler L.G., Zavarykina T.M., Kashcheev V.V. (2010). Biomarkers of oxidative stress and smoking in cancer patients. J. Cancer Res. Ther..

[29] Arathi A., D’Souza B., Sayanthan M., Raksha S., Buthesh G.A., Jisha K., Hegde M.C., D’Souza V. (2010). Department Salivary malondialdeyde and antioxidant status in oral squamous cell carcinoma patients and smokers. Biomed. Res..

[30] Barut O., Vural P., Şirin Ş., Aydin S., Dizdar Y. (2012). The oxidant/antioxidant status and cell death mode in oral squamous cell carcinoma. Acta Odontol. Scand..

[31] Ramya R., Prakash S., Sudha S. (2011). Assessment of Serum Malondialdehyde in Oral Squamous Cell Carcinoma patients and its association with tobacco habits. J. Pharm. Biomed. Sci..

[32] Srivastava K.C., Austin R.D., Shrivastava D., Sethupathy S., Rajesh S. (2012). A Case control study to evaluate oxidative stress in plasma samples of oral malignancy. Contemp. Clin. Dent..

[33] Shetty S.R., Babu S., Kumari S., Shetty P., Hegde S., Castelino R. (2014). Status of salivary lipid peroxidation in oral cancer and precancer. Indian J. Med. Paediatr. Oncol..

[34] Nath A., Anand V., Anshu A.K., Rashmi T., Singh J., Jain P., Sinha R., Kumar S. (2014). Significantly high levels of estrogen and MDA together induce tumor progression in Oral squamous cell carcinoma. IOSR J. Environ. Sci. Toxicol. Food Technol..

[35] Rasool M., Khan S.R., Malik A., Khan K.M., Zahid S., Manan A., Qazi M.H., Naseer M.I. (2014). Comparative Studies of Salivary and Blood Sialic Acid, Lipid Peroxidation and Antioxidative Status in Oral Squamous Cell Carcinoma (OSCC). Pak. J. Med. Sci..

[36] Ganesan A., Kumar G. (2014). Assessment of lipid peroxides in multiple biofluids of leukoplakia and oral squamous cell carcinoma patients-a clinico-biochemical study. J. Clin. Diagn. Res..

[37] Malik U.U., Siddiqui I.A., Hashim Z., Zarina S. (2014). Measurement of serum paraoxonase activity and MDA concentrations in patients suffering with oral squamous cell carcinoma. Clin. Chim. Acta.

[38] Huo W., Li Z.-M., Pan X.-Y., Bao Y.-M., An L.-J. (2014). Antioxidant enzyme levels in pathogenesis of oral squamous cell carcinoma (OSCC). Drug Res..

[39] Bhat V.S., Nayak K.R., Kini S., Bhat S.P. (2016). Assessment of serum antioxidant levels in oral and oropharyngeal carcinoma patients. Internet J. Pathol. Lab. Med..

[40] Rai S., Sharma A., Ranjan V., Misra D., Panjwani S. (2015). Estimation of serum antioxidant enzymes in histopathological grades of oral leukoplakia, oral submucous fibrosis, and oral cancer: A clinicopathologic study. J. Indian Acad. Oral Med. Radiol..

[41] Kaur J., Politis C., Jacobs R. (2016). Salivary 8-hydroxy-2-deoxyguanosine, malondialdehyde, vitamin C, and vitamin E in oral pre-cancer and cancer: Diagnostic value and free radical mechanism of action. Clin. Oral Investig..

[42] Shankarram V., Narayanan M.L., Sudhakar M.U., Moses M.J., Selvan M.T., Parthiban MD S.S. (2015). Detection of Oxidative Stress in Periodontal Disease and Oral Cancer. Biomed. Pharmacol. J..

[43] Misra D., Rai S., Panjwani S., Sharma A., Singh N. (2016). Role of antioxidants as a stress factor for potentially malignant, malignant disorders and healthy individuals: A correlative study. J. Dr. NTR Univ. Health Sci..

[44] Nyamati S.B., Annapoorna H.B., Tripathi J., Sinha N., Roy S., Agrawal R. (2016). Evaluation of serum antioxidant enzymes in oral submucous fibrosis and oral squamous cell carcinoma: A clinical and biochemical study. J. Adv. Med. Dent. Sci. Res..

[45] Srivastava K.C., Austin R.D., Shrivastava D. (2016). Evaluation of oxidant-antioxidant status in tissue samples in oral cancer: A case control study. Dent. Res. J..

[46] Verma S., Saxena R., Siddiqui M.H., Santha K., Sethupathy S. (2017). Evaluation of CYP1B1 Expression, Oxidative Stress and Phase 2 Detoxification Enzyme Status in Oral Squamous Cell Carcinoma Patients. J. Clin. Diagn. Res..

[47] Madhulatha G., Venkateswarlu N., Das S.V. (2017). Estimations of various antioxidants in oral cancer patients in comparison with smokers and non-smokers—A biochemical study. Int. J. Res. Med. Sci..

[48] Banerjee S., Mukherjee S., Mitra S., Singhal P. (2017). Altered expression of mitochondrial antioxidants in oral squamous cell carcinoma. J. Oral Sci..

[49] Basu S. (2018). Medpulse International Journal of Biochemistry.

[50] Sabarathinam J., Selvaraj J., Devi S. (2019). Estimation of Levels of Glutathione Peroxidase (Gpx), Malondialdehyde (Mda), Tumor Necrosis Factor Alpha (Tnf Alpha) and Alpha Feto Protein (Afp) In Saliva of Potentially Malignant Disorders and Oral Squamous Cell Carcinoma. Biomed. Pharmacol. J..

[51] Babiuch K., Bednarczyk A., Gawlik K., Pawlica-Gosiewska D., Kęsek B., Darczuk D., Stępień P., Chomyszyn-Gajewska M., Kaczmarzyk T. (2019). Evaluation of enzymatic and non-enzymatic antioxidant status and biomarkers of oxidative stress in saliva of patients with oral squamous cell carcinoma and oral leukoplakia: A pilot study. Acta Odontol. Scand..

[52] Shahi Y., Samadi F.M., Mukherjee S. (2020). Plasma lipid peroxidation and antioxidant status in patients with oral precancerous lesions and oral cancer. Oral Sci. Int..

[53] Oswal R.G., Nandan K.R., Prashant D.I.G.M. (2020). Evaluation of serum antioxidant enzymes in oral submucous fibrosis and oral squamous cell carcinoma: A clinical and biochemical study. Eur. J. Mol. Clin. Med..

[54] Abdelkawy M., El Refai S., Shaker O.G., Elbattawy W. (2020). Malondialdehyde and Nitrous Oxide as Salivary Biomarkers for Different Oral Lesions. Adv. Dent. Res..

[55] Ohkawa H., Ohishi N., Yagi K. (1979). Assay for lipid peroxides in animal tissues by thiobarbituric acid reaction. Anal. Biochem..

[56] Suematsu T., Kamada T., Abe H., Kikuchi S., Yagi K. (1977). Serum lipoperoxide level in patients suffering from liver diseases. Clin. Chim. Acta.

[57] Yagi K. (1987). Lipid peroxides and human diseases. Chem. Phys. Lipids.

[58] Donnan S.K. (1950). The Thiobarbituric Acid Test Applied to Tissues from Rats Treated in Various Ways. J. Biol. Chem..

[59] Buege J.A., Aust S.D. (1978). Microsomal lipid peroxidation. Methods in Enzymology.

[60] Draper H.H., Hadley M. (1990). Malondialdehyde determination as index of lipid Peroxidation. Methods in Enzymology.

[61] Bergmeyer H.-U. (1974). Methods of Enzymatic Analysis V2.

[62] Jain S.K., McVie R., Duett J., Herbst J.J. (1989). Erythrocyte membrane lipid peroxidation and glycosylated hemoglobin in diabetes. Diabetes.

[63] Stocks J., Dormandy T.L. (1971). The Autoxidation of Human Red Cell Lipids Induced by Hydrogen Peroxide. Br. J. Haematol..

[64] Valenzuela A. (1991). The biological significance of malondialdehyde determination in the assessment of tissue oxidative stress. Life Sci..

[65] Shilpasree A.S., Kumar K., Itagappa M., Ramesh G. (2013). Study of oxidative stress and antioxidant status in oral cancer patients. Int. J. Oral Maxillofac. Pathol..

[66] Satoh K. (1978). Serum lipid peroxide in cerebrovascular disorders determined by a new colorimetric method. Clin. Chim. Acta..

[67] Thomas S.A., Sethupathy S. (2015). Evaluation of Oxidative Stress in Patients with Oral Squamous Cell Carcinoma. Int. J. Pharm. Bio. Sci..

[68] Mahfouz M.O., Hariprasad C.H., Shaffie I.A., Sadasivudu B. (1986). Serum Malondialdehyde levels in myocardial infarction and chronic renal failure. IRCS Med. Sci..

[69] Sinnhuber R.O., Yu T.C., Yu T.C. (1958). Characterization of the Red Pigment Formed in the 2-thiobarbituric Acid Determination of Oxidative Rancidity b. J. Food Sci..

[70] Gavino V.C., Miller J.S., Ikharebha S.O., Milo G.E., Cornwell D.G. (1981). Effect of polyunsaturated fatty acids and antioxidants on lipid peroxidation in tissue cultures. J. Lipid Res..

[71] Ogura R., Sakanashi T., Nagata O., Sugiyama M., Kajiyama K., Nakagawa T., Shin G., Hidaka T. (1987). Assay for lipid peroxide content in mitochondria by the thiobarbituric acid reaction. Kurume Med. J..

[72] Nair V., Turner G.A. (1984). The thiobarbituric acid test for lipid peroxidation: Structure of the adduct with malondialdehyde. Lipids.

[73] Sobin L.H.W.C. (1997). International Union Against Cancer (UICC): TNM Classification of Malignant Tumours.

[74] Bryne M., Koppang H.S., Lilleng R., Stene T., Bang G., Dabelsteen E. (1989). New malignancy grading is a better prognostic indicator than Broders’ grading in oral squamous cell carcinomas. J. Oral Pathol. Med..

[75] Akhter M., Hossain S., Rahman Q.B., Molla M.R. (2011). A study on histological grading of oral squamous cell carcinoma and its co-relationship with regional metastasis. J. Oral Maxillofac. Pathol..

[76] Anneroth G., Batsakis J., Luna M. (1987). Review of the literature and a recommended system of malignancy grading in oral squamous cell carcinomas. Scand. J. Dent. Res..

[77] Mayes P.A., Murray R.K., Granner D.K. (2000). Harper’s Biochemistry.

[78] Klaunig J.E., Kamendulis L.M., Hocevar B.A. (2010). Oxidative Stress and Oxidative Damage in Carcinogenesis. Toxicol. Pathol..

[79] Tseng S.-K., Chang M.-C., Su C.-Y., Chi L.-Y., Chang J.Z.-C., Tseng W.-Y., Yeung S.-Y., Hsu M.-L., Jeng J.-H. (2012). Arecoline induced cell cycle arrest, apoptosis, and cytotoxicity to human endothelial cells. Clin. Oral Investig..

[80] Salzman R., Pácal L., Tomandl J., Kanková K., Tóthová E., Gál B., Kostrica R., Salzman P. (2009). Elevated malondialdehyde correlates with the extent of primary tumor and predicts poor prognosis of oropharyngeal cancer. Anticancer Res..

[81] Fleming I.D., Cooper J.S., Henson D.E., Hutter R.V., Kennedy B.J., Murphy G., O’Sullivan B., Sobin L.H., Yarbro J.W. (1997). AJCC Cancer Staging Manual.

[82] Bennardo L., Bennardo F., Giudice A., Passante M., Dastoli S., Morrone P., Provenzano E., Patruno C., Nisticò S.P. (2021). Local Chemotherapy as an Adjuvant Treatment in Unresectable Squamous Cell Carcinoma: What Do We Know So Far?. Curr. Oncol..

[83] Pentangelo G., Nisticò S.P., Provenzano E., Cisale G.Y., Bennardo L. (2021). Topical 5% Imiquimod Sequential to Surgery for HPV-Related Squamous Cell Carcinoma of the Lip. Medicina.

